# Unravelling the distinct contribution of cell shape changes and cell intercalation to tissue morphogenesis: the case of the *Drosophila* trachea

**DOI:** 10.1098/rsob.200329

**Published:** 2020-11-25

**Authors:** Sandra Casani, Jordi Casanova, Marta Llimargas

**Affiliations:** 1 Institut de Biologia Molecular de Barcelona, CSIC, Parc Científic de Barcelona, Baldiri Reixac, 10-12, 08028 Barcelona, Catalonia, Spain; 2 Institute for Research in Biomedicine (IRB Barcelona), The Barcelona Institute of Science and Technology (BIST), Baldiri Reixac 10, 08028 Barcelona, Catalonia, Spain

**Keywords:** *Drosophila*, morphogenesis, dorsal branch extension, cell intercalation, cell elongation

## Abstract

Intercalation allows cells to exchange positions in a spatially oriented manner in an array of diverse processes, spanning convergent extension in embryonic gastrulation to the formation of tubular organs. However, given the co-occurrence of cell intercalation and changes in cell shape, it is sometimes difficult to ascertain their respective contribution to morphogenesis. A well-established model to analyse intercalation, particularly in tubular organs, is the *Drosophila* tracheal system. There, fibroblast growth factor (FGF) signalling at the tip of the dorsal branches generates a ‘pulling’ force believed to promote cell elongation and cell intercalation, which account for the final branch extension. Here, we used a variety of experimental conditions to study the contribution of cell elongation and cell intercalation to morphogenesis and analysed their mutual requirements. We provide evidence that cell intercalation does not require cell elongation and vice versa. We also show that the two cell behaviours are controlled by independent but simultaneous mechanisms, and that cell elongation is sufficient to account for full extension of the dorsal branch, while cell intercalation has a specific role in setting the diameter of this structure. Thus, rather than viewing changes in cell shape and cell intercalation as just redundant events that add robustness to a given morphogenetic process, we find that they can also act by contributing to different features of tissue architecture.

## 1. Introduction

Cell intercalation is a key mechanism underlying tissue remodelling during morphogenesis. Through this mechanism, cells exchange positions in a spatially oriented manner in diverse processes, ranging from convergent extension in embryonic gastrulation to the formation of tubular organs. In the cases studied, cell intercalation is coupled to junction remodelling, thought to be caused by anisotropic changes of tension along the different surfaces of the cell, which are under strict genetic control (for reviews, see [1–6]). However, given the co-occurrence of cell intercalation and cell shape changes, it is often difficult to ascertain their respective contribution to morphogenesis. The analysis of a diversity of morphogenetic events involving these cell behaviours in different contexts is therefore critical to shed light into their specific roles in morphogenesis and to unveil the generalities and specificities of tissue remodelling.

The *Drosophila* tracheal system is a widely used model in which to study intercalation, particularly in tubular organs [6,7]. More precisely, the analysis of cell intercalation in the dorsal branches of the trachea has provided a good description of the different steps of cell intercalation and elucidation of the genetic control of this process [8–12]. In this regard, a cell intercalation mechanism has been proposed. Briefly, activation of the fibroblast growth factor (FGF) receptor Breathless (Btl) at the tip of the tracheal branches by its ligand Branchless (Bnl), which is secreted by nearby cells, induces an attraction of the tracheal cells towards neighbouring cells [13]. This attraction generates a ‘pulling’ force believed to promote a change in cell shape (cell elongation) and to drive the rearrangement of cells from a side-by-side to an end-to-end arrangement (cell intercalation), a process accompanied by the conversion of intercellular to autocellular adherens junctions (AJs) [9,11].

The combined effects of cell elongation and cell intercalation account for the final lengthening of the dorsal branches [9]. However, the extent to which each event contributes to dorsal branch extension and how the two events are related are unknown. In fact, it is quite widely assumed that cell elongation triggers cell intercalation [5]. Here, we used different experimental conditions to investigate cell elongation and cell intercalation during dorsal branch extension and analysed their mutual requirements. We provide evidence that the two cell behaviours, responding to the same attracting signal, are controlled by independent but simultaneous mechanisms. Moreover, we show that cell elongation alone is sufficient to account for full dorsal branch extension, a morphogenetic event that can occur even when cell intercalation is impaired. Conversely, we demonstrate that cell intercalation plays a specific role in determining the diameter of dorsal branches. Thus, rather than viewing changes in cell shape and cell intercalation as just redundant events that add robustness to a given morphogenetic process, we find that they can also act by contributing to different features of tissue architecture.

## 2. Results and discussion

### 2.1. Cell intercalation is not required for full dorsal branch extension

**Figure 1.**
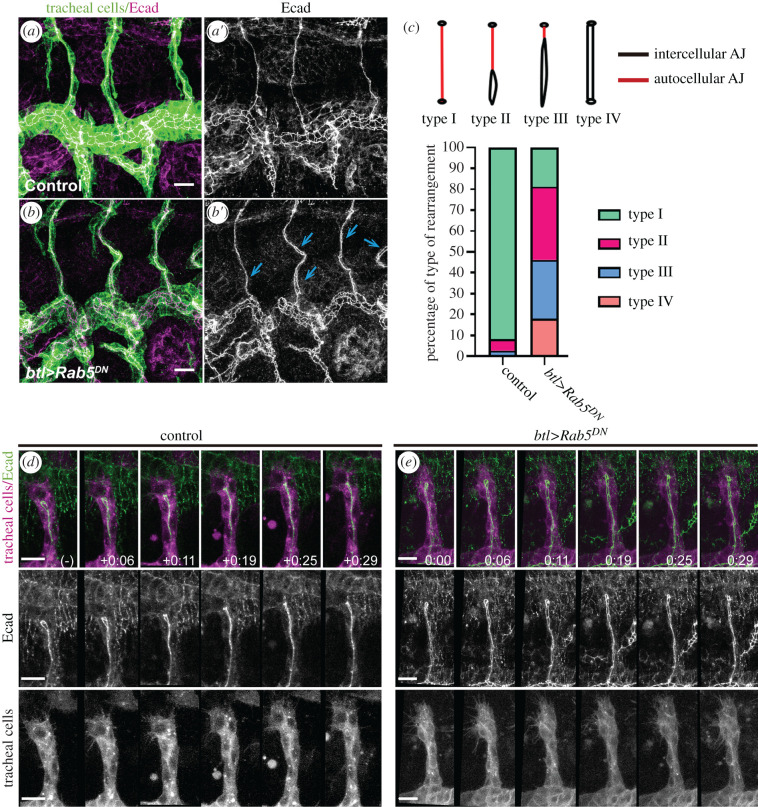
Effects of Rab5 downregulation on cell intercalation. (*a,b*) Confocal projections showing lateral views of btl > srcGFP control embryos (*a*) and btl > srcGFP + Rab5^DN^ embryos (*b*) at stage 16, both incubated at 29°C, stained for Ecad (magenta) and GFP (green). Note the presence of non-intercalated regions in the mutants (blue arrows in *b*′). (*c*) Type of junctional rearrangements observed in the dorsal branches. Type I corresponds to completely autocellular (intercalated) junctions, Type II to mostly autocellular junctions, Type III to mostly intercellular (non-intercalated) junctions and Type IV to completely intercellular junctions. Quantification of each type of cell rearrangement observed in the indicated genotypes, shown as a percentage of each type (control *n* = 116; Rab5^DN^ *n* = 126 cell rearrangements). (*d*,*e*) Stills from time-lapse movies of a dorsal branch of a btl > mCherryCAAX; EcadGFP control embryo (*d*) and a btl > mCherryCAAX + Rab5^DN^; EcadGFP mutant embryo (*e*). mCherryCAAX (magenta) marks the contour of the cells and EcadGFP (green) marks the AJs. Scale bar, 10 µm.

We and others have previously described genetic mechanisms that interfere with cell intercalation in the dorsal branches [9,11,12]. In particular, we showed that Rab5-mediated endocytosis of Ecad plays a key role in cell intercalation and that the expression of a dominant negative form of Rab5 (Rab5^DN^) leads to a failure of this process [12]. Hence, we used this experimental condition to study the effects of defective cell intercalation on dorsal branch extension. The expression of Rab5^DN^ showed a fully penetrant phenotype in cell intercalation (defects in all dorsal branches) with variable expressivity, according to the four-point scale we previously devised [12]. Most cells kept complete (Type IV) or partial intercellular (non-intercalated) contacts (Type II and III) at stage 16, when the intercellular junctions of wild-type cells had been replaced by autocellular (intercalated) ones (Type I) (figure 1*a–c*; electronic supplementary material, figure S1A,B). We also corroborated these defects in cell intercalation by *in vivo* life imaging, which also revealed that intercalation defects were more frequent in cells of the distal portion of the dorsal branches (figure 1*d,e*; electronic supplementary material, movies S1 and S2).

**Figure 2.**
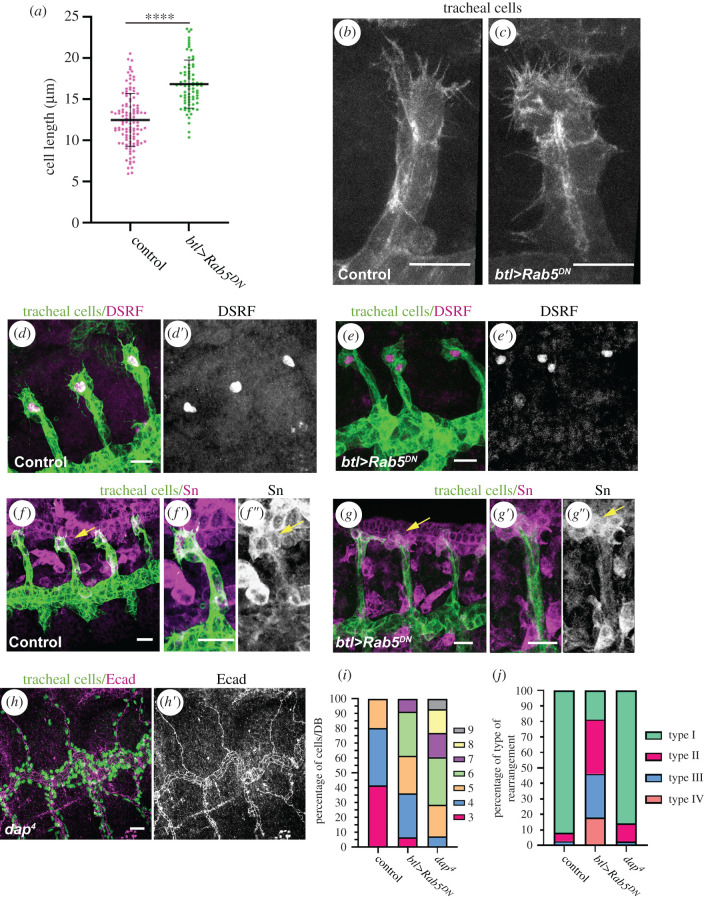
Effects of Rab5 downregulation on cell elongation and dorsal branch formation. (*a*) Scatter plot quantifying the cell length in the indicated genotypes (control *n* = 117; Rab5^DN^ *n* = 75 cells). Rab5^DN^ non-intercalated cells are significantly longer than the control cells. ****p < 0.0001 obtained by unpaired two-tailed Student's *t-*test applying Welch's correction. (*b*,*c*) Stills from *in vivo* experiments in btl::MoeGFP control (*b*) and btl::MoeGFP;btl > Rab5^DN^ mutant (*c*) embryos showing tip cells exhibiting filopodia. (*d*–*g*) Confocal projections showing lateral views of btl > srcGFP control embryos (*d*,*f*) and btl > srcGFP + Rab5^DN^ mutant embryos (*e*,*g*). Stage 14/15 embryos were stained with DSRF (magenta, *d*,*e*), Sn (magenta *f*,*g*) and GFP (green). (*h*) Confocal projection showing a lateral view of a *dap^4^* mutant stained for Ecad (magenta) and Trh (green) to show the tracheal nuclei. (*i*) Stack bars graphic showing the distinct percentages of the number of stalk cells per dorsal branch in the indicated genotypes (control, *n* = 41; Rab5^DN^, *n* = 47; and *dap^4^*, *n* = 56 dorsal branches). (*j*) Quantification of the type of cell rearrangement observed in the indicated genotypes, shown as a percentage of each type (control *n* = 116; Rab5^DN^ *n* = 126; dap^4^ *n* = 352 cell rearrangements). Scale bar, 10 µm.

However, in spite of these strong defects in cell intercalation, all Rab5^DN^ dorsal branches reached the midline in a simultaneous manner and fused with the contralateral branches (figure 1*b*; electronic supplementary material, figure S1A,B). We found that, indeed, dorsal branches were able to fully extend due to increased cell elongation. In particular, we observed that cells unable to intercalate in Rab5^DN^ conditions underwent an extra elongation when compared with control cells (16.85 ± 2.92 µm cell length in Rab5^DN^ non-intercalated cells versus 12.49 ± 3.21 µm in wild-type cells) (figure 2*a*) and to intercalated cells in Rab5^DN^ conditions (15.79 ± 1.61 µm cell length in Rab5^DN^ non-intercalated cells versus 9.466 ± 0.51 µm in Rab5^DN^ intercalated cells; *n* = 8 dorsal branches analysed; see Material and methods for measuring procedure; electronic supplementary material, figure S1C,D).

To better characterize the Rab5^DN^ tracheal cells and discard a general disruption, we first assessed that their overall apicobasal polarity was not affected as judged by the proper localization of the apical determinant Crumbs (Crb) and the septate junction marker Megatrachea (Mega) (electronic supplementary material, figure S2A,B,D,E). We also examined the activity of the Bnl/Btl pathway in Rab5^DN^ dorsal branches, as this pathway provides the pulling force for dorsal branch extension and cell intercalation [9,11–13]. The DSRF transcription factor and the Fascin/Singed actin-cross-linker, well-known targets of the Bnl/Btl pathway [14–16] were normally expressed at the tip of the Rab5^DN^ dorsal branches (figure 2*d*–*g*). In addition, we observed thin cell extensions in the tip cells corresponding to filopodia, as in the control (figure 2*b*,*c*). Therefore, as indicated by the ability of the Rab5^DN^ dorsal branches to fully extend, their tip cells receive signals from the Bnl/Btl signalling pathway and elicit its responses.

As we previously reported, Rab5^DN^ expression also increased the number of cells allocated to each dorsal branch [12] (figure 2*i*). However, an increased number of cells in these branches did not appear to cause the intercalation defects, as *dacapo* (*dap*) mutant embryos, which undergo an extra round of cell division [17,18], showed more cells forming the dorsal branches than upon Rab5^DN^ expression (figure 2*i*) and yet no significant defects in cell intercalation (figure 2*h*,*j*).

All together, these results reveal that dorsal branches can fully extend when intercalation is defective, thereby suggesting that cell intercalation is not a requisite for dorsal branch extension. Indeed, regarding defective cell intercalation, we show that complete dorsal branch extension is achieved by extra elongation of the cells, further indicating that, in addition, elongation does not require cell intercalation.

### 2.2. Cell intercalation does not require cell elongation

**Figure 3.**
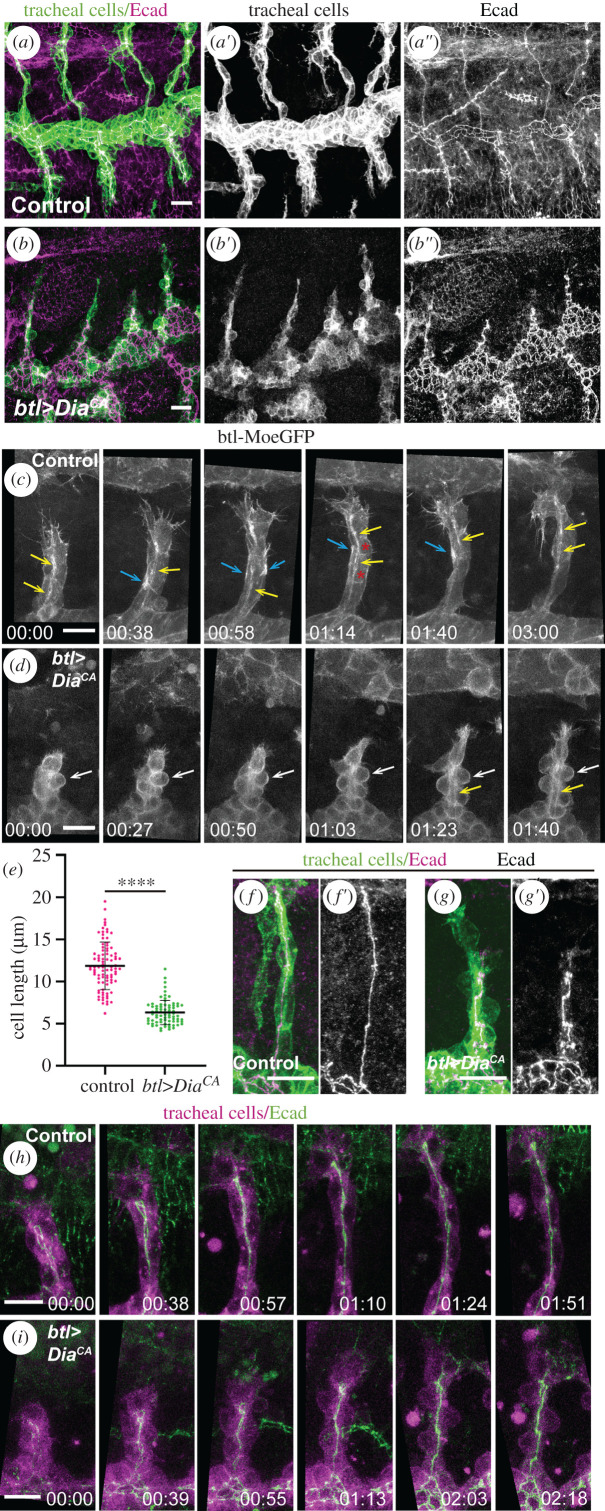
Effects of constitutive activation of Dia on cell intercalation and cell elongation. (*a*,*b*) Confocal projections showing lateral views of btl > srcGFP control embryos (*a*) and btl > srcGFP + Dia^CA^ embryos (*b*), both incubated at 25°C, stained for Ecad (magenta) and GFP (green). Dorsal branches in control trachea extend until they reach the midline and cells elongate and intercalate (*a*). By contrast, in Dia^CA^ mutants, cells do not elongate and branches do not extend (*b*). (*c*,*d*) Stills from *in vivo* experiments showing actin dynamics in btl::MoeGFP control (*c*) and btl::MoeGFP;btl > Dia^CA^ mutant (*d*) dorsal branches. Differences in localization and flow dynamics of MoeGFP can be observed between Dia^CA^ and the control. Control trachea (*c*) show an apical flow (yellow arrow) and highly dynamic actin flows localized at the basal membrane (blue arrow); perpendicular actin flows are also sporadically observed (red asterisks). Dia^CA^ mutants (*d*) show a more ubiquitous GFP signal, highlighting the rounded cell shape (white arrows). MoeGFP apical localization was observed later (yellow arrow) and it was less dynamic than in the control. Time is shown in hh:mm format. (*e*) Scatter plot quantifying cell length in the indicated genotypes at stage 16 (control *n* = 89; Dia^CA^ *n* = 75 cells). ****p < 0.0001 obtained by unpaired two tail Student's *t-test* applying Welch's correction. (*f*–*g*) Details of a single tracheal metamere, stained for Ecad (magenta) and GFP (green), showing completed intercalation in control (*f*) and Dia^CA^ (*g*) conditions. Note the formation of autocellular adherens junctions indicating intercalation in Dia^CA^ conditions in spite of the abnormal alignment of the cells, probably due to the abnormal cell shape. (*h*,*i*) Stills from time-lapse movies of a dorsal branch of a btl > mCherryCAAX; EcadGFP control embryos (*h*) and a btl > mCherryCAAX + Dia^CA^; EcadGFP mutant embryo (*i*). Ecad (green) marks AJs and mCherryCAAX (magenta) marks cell membranes. Time is shown in hh:mm format. Scale bars, 10 µm.

To further assess the relationship between cell elongation and cell intercalation, we sought to identify a condition in which cell shape changes were impaired. We found that moderate levels of a constitutively active form of Diaphanous (Dia, UAS-Dia^CA^) in the tracheal system altered cell shape (figure 3*a*,*b*). Dia encodes a protein of the Formin family that contributes to the nucleation and elongation of F-actin filaments (reviewed in [19]). In agreement with this finding, we observed that tracheal expression of Dia^CA^, which lacks the predicted regulatory and autoinhibitory domains of the Dia protein, altered actin organization. In control tracheae, actin flows, as visualized by MoeGFP [20], were highly dynamic and transient in the basal membrane (blue arrow in figure 3*c*; electronic supplementary material, movie S3). In addition, we also observed occasional flows perpendicular to the length of the dorsal branch (asterisks in figure 3*c*), which may correspond to the junction between two neighbouring cells, and a thin but persistent signal at the apical cortex of the cells corresponding to continuous flows (yellow arrows in figure 3*c*). Conversely, in Dia^CA^ conditions, MoeGFP displayed a more ubiquitous, uniform and stable distribution of actin around the whole cell cortex (electronic supplementary material, movie S4) throughout the whole process of dorsal branch extension (white arrows in figure 3*d*), and we did not detect the typical flows seen in the control or a distinguishable signal at the apical cortex (yellow arrows in figure 3*d*).

**Figure 4.**
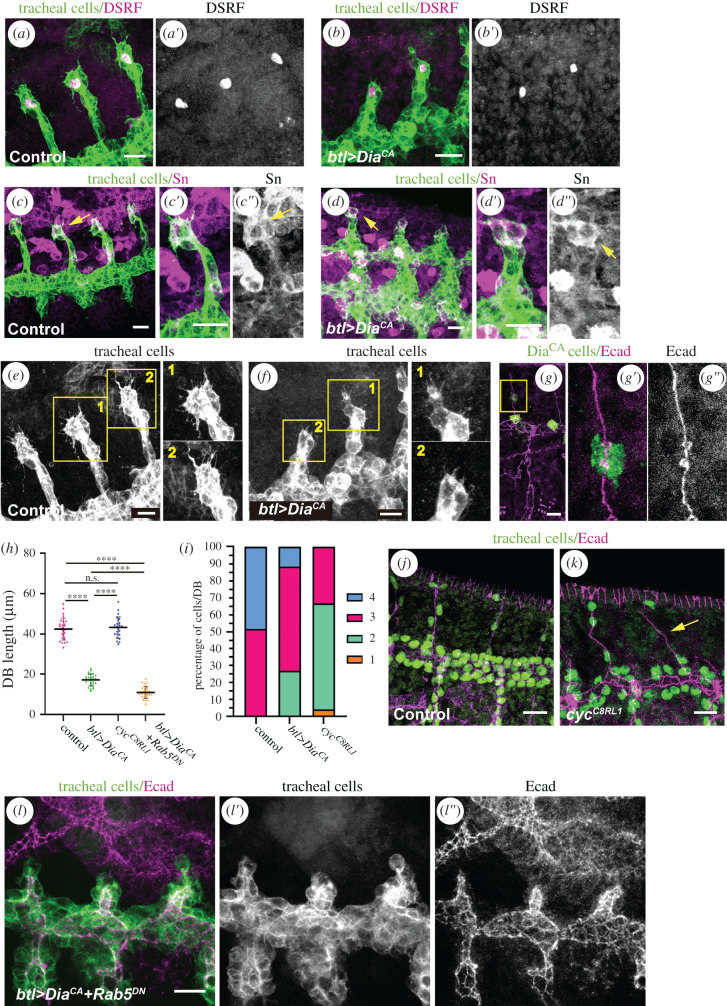
Effects of constitutive activation of Dia on dorsal branch formation and extension. (*a*–*d*) Confocal projections showing lateral views of btl > srcGFP control embryos (*a*,*c*) and btl > srcGFP + Dia^CA^ mutant embryos (*b*,*d*), incubated at 25°C. Stage 14/15 embryos were stained with DSRF (magenta, *a,b*), Sn (magenta *c*,*d*) and GFP (green). (*e*,*f*) Confocal projections of lateral views of dorsal branches showing tip cells exhibiting filopodia. Magnifications of btl > srcGFP control (*e*) and btl > srcGFP + Dia^CA^ mutants (*f*) are shown as indicated. (*g*) Confocal projection of a lateral view of a stage 15 embryo containing Dia^CA^ single-cell clones in the trachea marked with GFP (green). Several Dia^CA^ cell clones localize in distinct branches. (*g*′–*g*″) Detail of a Dia^CA^ single-cell clone in a dorsal branch, showing a rounded cell shape (*g*′) and short length compared to the neighbouring cells (*g*″). (*h*) Scatter plot quantifying dorsal branch length of the indicated genotypes (control *n* = 27; Dia^CA^ *n* = 26; *cycA n* = 23; Dia^CA^Rab5^DN^ *n* = 30 dorsal branches). **** *p* < 0.0001 obtained by a Dunnett's T3 *post hoc* multiple comparisons test performed after a Welch's ANOVA test. n.s., not significant. (*i*) Stacked bars graphic showing the percentage of the number of stalk cells per dorsal branch in the indicated genotypes (control *n* = 27; Dia^CA^ *n* = 26; *cycA n* = 23 dorsal branches). (*j*–*k*) Confocal projection showing a lateral view of a control (*j*) and a *cycA^C8LR1^* (*k*) embryo, stained for Ecad (magenta) and Trh (green). Note that a single cell forms the stalk of a completely extended dorsal branch in K (yellow arrow). (*l*) Confocal projection showing a lateral view of a stage 15 btl > srcGFP + Dia^CA^ + Rab5^DN^ mutant embryo stained for Ecad (magenta) and GFP (green). Dia^CA^+Rab5^DN^ causes an additive effect, as cells are rounded and do not undergo cell intercalation. Scale bars, 10 µm.

Moderate levels of Dia^CA^ gave rise to rounded cells that did not elongate (6.33 ± 1.44 µm in Dia^CA^ cells versus 11.89 ± 2.80 µm in wild-type cells at stage 16; figure 3*e*). In the wild-type, cells become significantly more elongated as development proceeds (7.11 ± 1.70 µm at stage 14 and 11.89 ± 2.80 µm at stage 16), whereas in Dia^CA^ conditions, the cells did not undergo this remarkable cell elongation (5.64 ± 1.28 µm at stage 14 and 6.33 ± 1.44 µm at stage 16 in Dia^CA^ cells; figure 3*e*). This phenotype was fully penetrant, with all branches displaying rounded cells. However, in spite of not elongating, the rounded cells in most Dia^CA^ dorsal branches were still able to change their intercellular AJs to autocellular ones and to intercalate (70.6% of DB showed normal intercalation, *n* = 86; figure 3*f*,*g*). *In vivo* time-lapse analyses confirmed these observations and showed that cells became rounded from the beginning of branch extension and remained rounded throughout intercalation, as reflected by the curved shape of basal membranes (figure 3*h*,*i*; electronic supplementary material, movie S5). *In vivo* analysis also showed that Dia^CA^ tracheal cells were still able to display movements (electronic supplementary material, movie S5) in spite of being unable to elongate, pointing to a specific effect on cell elongation. While the Dia^CA^ cells did not elongate, we assessed that they preserved their apicobasal polarity (electronic supplementary material, figure S2A,C,D,F) and responded properly to the Bnl/Btl pathway, as DSRF and Fascin/Singed were normally expressed and filopodia were present in the tip cells of the Dia^CA^ dorsal branches (figure 4*a*–*f*; electronic supplementary material, movies S3 and S4). Therefore, the lack of cell elongation in Dia^CA^ is not due to a failure of Btl signalling. We note that while moderate levels of constitutive activation of Dia disturbed cell elongation but not cell intercalation, stronger conditions, such as expression of the UAS-Dia^CA^ construct at 29°C, altered the overall tracheal morphology, impinging on both cell elongation and intercalation (electronic supplementary material, figure S3A–E).

Finally, a clonal analysis showed the effect of Dia^CA^ on cell shape to be cell autonomous. Dia^CA^ single-cell clones displayed a rounded shape, without affecting the ability of neighbouring cells to elongate or intercalate (figure 4*g*). Hence, our results reveal that cell intercalation can proceed in the absence of cell elongation, suggesting once again these two processes are separable events.

Our results also provide further evidence that cell elongation, and not cell intercalation, is the main contributor to dorsal branch extension. Indeed, Dia^CA^ dorsal branches were much shorter and did not reach the dorsal midline (17.21 ± 2.98 µm in Dia^CA^ versus 42.42 ± 5.44 µm in the wild-type; figure 4*h*). Detailed analysis of the Dia^CA^ dorsal branches also showed a decrease in the number of cells compared to the wild-type (figure 4*i*). This observation raised the possibility that the shorter length of the Dia^CA^ dorsal branches might be due to their reduced cell number. However, previous studies in *cycA* mutant embryos, which fail to have the last postblastodermic mitosis and thus have half the regular number of cells, already showed that the tracheal branches reach the length of the wild-type structures [21]. We also examined the dorsal branches of *cycA* mutant embryos (figure 4*h*–*k*) and found that they reached the length of wild-type ones even when almost the entire length of the branches was contributed by a single cell (arrow in figure 4*k*). This observation unveils the tremendous elongation capacity of tracheal cells, which can on its own account for the total length of the dorsal branches.

The above experiments indicate that cells in the dorsal branches elongate and intercalate through different but simultaneous events that are independently impaired in the Rab5^DN^ and in Dia^CA^ conditions, respectively. Indeed, the combination of the two mutant conditions gave rise to an additive phenotype, as these dorsal branches displayed rounded cells like Dia^CA^ cells and also the same intercalation defects as those observed in Rab5^DN^ cells (figure 4*l*). We also measured the length of the dorsal branches of Rab5^DN^Dia^CA^ double mutants, which were on average 6 µm shorter than those of Dia^CA^ mutants (11.34 ± 3.59 µm versus 17.21 ± 2.98 µm, respectively). These results suggest that, in the absence of cell elongation, cell intercalation confers only approximately 14% of the normal length of the dorsal branches (42.42 ± 5.44 µm in the wild-type; figure 4*h*). Nevertheless, we emphasize that this contribution of cell intercalation appears to be dispensable if cells are able to elongate, since, as shown above, dorsal branches can fully extend in Rab5^DN^ mutants, which show cell elongation but impaired cell intercalation.

### 2.3. Cell intercalation and tube diameter

**Figure 5.**
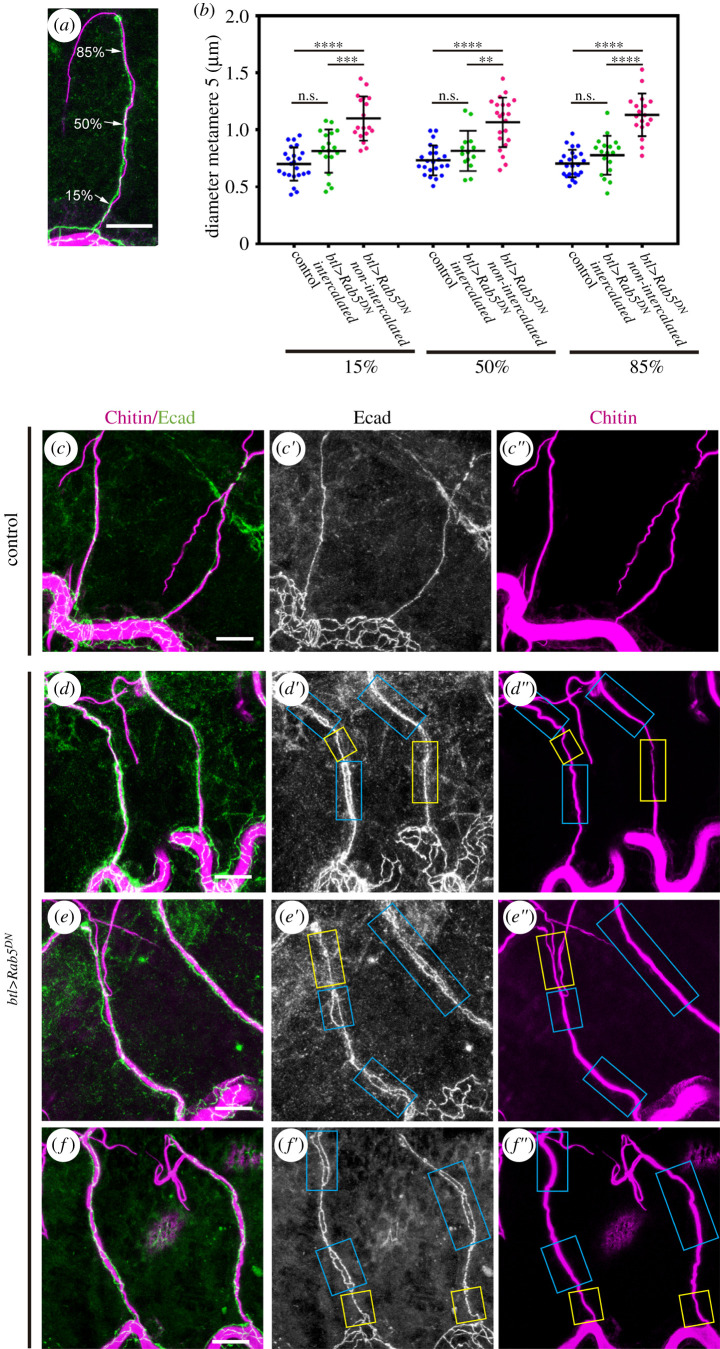
Effects of Rab5 downregulation on branch diameter. (*a*) Confocal projection of a lateral view of a stage 16 embryo showing the dorsal branch of tracheal metamere 5 stained for Ecad (green) and CBP (magenta) to visualize the lumen. Tube diameter was measured at position 15%, 50% and 85% of the length of the branch. (*b*) Scatter plot quantifying dorsal branch diameter at the positions indicated in the indicated genotypes (control, *n* = 23; Rab5^DN^, *n* = 36 dorsal branch 5). For Rab5^DN^ branches, we distinguish whether the region measured shows an intercalated or non-intercalated pattern. ****p-value < 0.0001, ***p-value < 0.001, **p-value < 0.01 obtained by Dunnett's T3 multiple comparisons *post hoc* tests performed after Welch's ANOVA test. n.s. not significant. (*c*–*f*) Confocal projections of lateral views of stage 16 embryos showing the metamere 4 and 5 dorsal branches stained for Ecad (green) and CBP (magenta). Note the smooth and homogeneous aspect of the tube diameter along the dorsal branch length in the control (*c*). By contrast, in Rab5^DN^ mutants (*d*–*f*), non-intercalated regions (blue boxes) are linked to slightly wider tubes and intercalated regions (yellow boxes) to thinner tube diameters. Scale bars, 10 µm.

Our results suggested an apparently unnecessary contribution of cell intercalation to dorsal branch extension. Therefore, we examined whether cell intercalation contributes to other features of dorsal branch morphogenesis. In particular, since cell intercalation leads to a single cell instead of two cells contributing to the lumen circumference of the dorsal branches, we addressed whether cell intercalation might be a factor in determining their tube diameter. To this end, we measured the lumen diameter at three points along the dorsal branches at positions of 15%, 50% and 85% of their length, 0% and 100% being the most proximal and distal positions, respectively (figure 5*a*). We measured the lumen diameter for the wild-type and Rab5^DN^ dorsal branch of the tracheal metamere 5 and for the latter, we distinguished whether the positions corresponded to intercalated or non-intercalated segments of the dorsal branch. The lumen of the non-intercalated regions of Rab5^DN^ dorsal branches were significantly wider than that of the wild-type branches (figure 5*c*–*f*) at all three positions (0.71 ± 0.027 µm on average in the wild-type versus 1.1 ± 0.045 µm in non-intercalated regions in Rab5^DN^, a 47% increase, figure 5*b*). Conversely, the lumen of wild-type dorsal branches and that of the intercalated regions of the Rab5^DN^ ones had a much more similar diameter (0.71 ± 0.027 µm on average in the wild-type versus 0.79 ± 0.045 µm in intercalated regions in Rab5^DN^, only a 11% increase; figure 5*b*).

While Rab5 activity might modulate tube diameter in various ways, the difference in lumen diameter between Rab5^DN^ non-intercalated and intercalated regions strongly links cell intercalation and branch diameter. In addition, these results are consistent with the findings of previous experiments showing that lumen width can be established autonomously by a single wild-type cell in a mutant tracheal branch [22]. Moreover, these same experiments by Forster *et al*. also indicated that the diameter of these wild-type cells spreads over a short range, thereby slightly extending into adjacent mutant cells. Thus, our results could account for (i) the widest diameter of the Rab5^DN^ branch lumen corresponding to the non-intercalated regions; and (ii) the slightly wider lumen diameter of the adjacent intercalated Rab5^DN^ regions than that of the wild-type intercalated control. Indeed, we observed a gradation in the width of the Rab5^DN^ branch lumen from non-intercalated to intercalated cells (for example, figure 5*d*–*f*), which could mechanically facilitate the smooth and homogeneous transition between lumina of distinct diameters.

To further confirm the link between cell intercalation and tube diameter in the trachea, we analysed other experimental conditions. On the one hand, we resorted to *sidekick* (*sdk*) mutants in which intercalation is also compromised [23], although to a lesser extent than in Rab5^DN^. In *sdk* embryos, we could also observe examples of wider tubes in non-intercalated regions (electronic supplementary material, figure S4D,E). On the other hand, we examined in detail the transverse connective, another tracheal branch that typically displays a mixture of intercalated and non-intercalated stretches [11]. Also in the transverse connective, we found a correlation between non-intercalated stretches and wider tubes (electronic supplementary material, figure S4A–C). Altogether these results support our conclusion that cell intercalation has a specific role in setting the branch diameter.

### 2.4. Cell elongation and cell intercalation are separable events

While cell elongation and cell intercalation in dorsal branches are ultimately triggered by the same attracting signal, as both are abrogated in *bnl* or *btl* mutants [9,11–13], our results show that one process can be impaired without interfering with the other, thereby suggesting that they are independently regulated events. We previously proposed a role for Rab5-mediated endocytosis in cell intercalation through the regulation of the intracellular trafficking of AJ molecules such as Ecad [12]. In the present study, we demonstrate that while impaired endocytosis prevents cell intercalation from progressing correctly (figure 1*b*,*c*), this same downregulation of Rab5 activity does not block the capacity of tracheal cells to elongate (figure 1*h*).

Conversely, we found that Dia^CA^, which stabilizes cortical actin in different contexts [24], has a strong effect on actin organization in tracheal cells and strongly impairs cell elongation, but instead allows these cells to intercalate. These results support the notion that cortical actin dynamics is a key player in the elongation of tracheal cells, which fits well with the observation that local changes in the composition or organization of the cortex can lead to cortical tension gradients that result in cell deformation [25]. Indeed, Dia is involved in the regulation of cell shape in various tissues; examples of this include dorsal closure and the formation of the segmental grooves [24,26–31].

Actin also affects AJs stability [32], so we would also expect Dia to participate in cell intercalation in dorsal branches, as it is the case in amnioserosa and epidermal cells, where Dia^CA^ stabilizes these junctions [24]. In agreement with this notion, we found that high levels of constitutively activated Dia not only impaired cell elongation but also affected cell intercalation (electronic supplementary material, figure S3). This observation suggests that actin regulation can affect both cell elongation and cell intercalation. However, distinct modes of actin modulation may exert different effects on one or the other event and with different strengths. Indeed, cells hold various actin pools, which, while interconnected, are specifically modulated and perform distinct cellular roles [33,34]. Similarly, a stronger or a distinct disruption of intracellular trafficking might also affect cell elongation. In this scenario, cell elongation and intercalation might be independently regulated in distinct manners and/or different degrees of actin polymerization and intracellular trafficking. In the present study, the use of particular mutant conditions has allowed us to impinge on one and not the other event, offering an ideal experimental setting in which to verify that cell elongation and cell intercalation in dorsal branches are independent events, and thus showing that cell elongation does not drive cell intercalation and that cells do not need to intercalate to elongate.

### 2.5. Contribution of cell elongation and cell intercalation to dorsal branch morphogenesis and beyond

The association of cell rearrangements and changes in cell shape is a common theme during tissue remodelling and is most exemplified in convergent extension [35]. Indeed, since cell intercalation was found to be associated with the elongation of many tissues in different organisms, it was proposed to have a major role in this process [36–38]. However, given the concomitant occurrence of cell shape changes and cell rearrangements, ascertaining the specific contribution of each event has been elusive. Previous work began to address this issue and, for example, the analysis of the morphogenesis of zebra fish revealed that extension could be regulated separately from convergence [39]. More recently, a similar analysis on the initial phase of germ band extension in the *Drosophila* embryo showed that mutations of patterning genes also allowed cell intercalation to be dissociated from cell shape changes [40]. Here, we have gone a step further and, by specifically impairing an actin regulator or a trafficking molecule, we dissociated cell intercalation and cell elongation in the formation of the dorsal branches of the *Drosophila* trachea. Moreover, our study also reveals that cell intercalation is dispensable to dorsal branch extension, although being dispensable does not necessarily imply that it does not contribute in the wild-type condition. However, we show that it is cell elongation what provides the major contribution to dorsal branch extension, as these structures can still fully extend when cell intercalation is impaired, thanks to extra elongation of their cells. Similarly, cell shape changes can also compensate for a failure of cell intercalation in the initial phase of *Drosophila* germ band extension [40].

While in several morphogenetic events distinct cell behaviours may act redundantly to ensure the robustness of the process, it is difficult to claim redundancy when one of the events, as it is the case of cell intercalation in the tracheae, appears to be dispensable for tissue extension. In this regard, we would like to suggest that, also in cases other than dorsal branches, the major role of cell intercalation may not be to add to tissue extension but rather to contribute to another morphogenetic event required for proper and functional tissue formation.

## 3. Material and methods

**Table:** 

UAS constructs	Chr	description	origin
UAS-Dia^CA^	III	UAS fused to a constitutively active form of the Dia protein	BDSC 27616
UAS-Rab5^DN^	III	UAS fused to a dominant negative form of the Rab5 protein	M. González-Gaitán
UAS-Dia^CA^, UAS-Rab5^DN^	III	recombinant stock of the two UAS fused to the indicated constructs	this study
UAS-GFP	III	UAS fused to a ubiquitous GFP	J. Solon
UAS-Dia^CA^, UAS-GFP	III	UAS-Dia^CA^ recombined with UAS-GFP	this study
UAS-Rab5^DN^, UAS-GFP	III	UAS-Rab5^DN^ recombined with UAS-GFP	this study
UAS-mCherryCAAX	II	UAS fused to the monomeric Cherry fluorescent protein targeted to CAAX motif that marks the cell membrane	BDSC 59021
UAS-mCherryCAAX; UAS-Dia^CA^	II; III	stock carrying two UAS fused to the indicated proteins	this study
UAS-mCherryCAAX; UAS-Rab5^DN^	II; III	stock carrying two UAS fused to the indicated proteins	this study
Gal4 drivers	Chr	description	origin
*btlGal4, UAS-SrcGFP*	II	*btlGal4* driver recombined with a UAS fused to SrcGFP that marks the cell membrane	Llimargas and Casanova labs
*btlGal4, DE-cadGFP*	II	*btlGal4* driver recombined with a knock-in allele of Ecad protein fused to GFP	Llimargas lab
*btlGal4; btl::MoeGFP*	II; III	*btlGal4* driver combined with *btl* promoter fused to a MoesinGFP construct	Llimargas and Casanova labs
*hsFLP^122^; btl::RFPMoe, btl FRT > y+ > FRT Gal4*	I; III	heat shock *Flipase* promoter activating *btlGal4* driver	M. Affolter
alleles	Chr	description	origin
*cycA^C8LR1^*/ TM3	III	amorphic allele	BDSC 6627
*dap^4^*/ CyO	II	amorphic allele	BDSC 6639
*sdk^MB05054^*	I	loss of function allele	BDSC 24603

Abbreviations used: Chr, chromosome/s; BDSC, Bloomington Drosophila Stock Center.[Table RSOB200329TB2]

**Table RSOB200329TB2:** 

primary antibodies			
name	generated in	Conc.	source/provided by
anti-Crb (Cq4)	mouse	1:10	DSHB
anti-DSRF	rabbit	1:500	produced by N. Martín in Casanova lab
anti-Ecad (DCAD2)	rat	1:100	DSHB
anti-Ecad	goat	1:600	Santa Cruz
anti-GFP	goat	1:300	ABCAM
anti-GFP	rabbit	1:300	Thermo Fisher
anti-Sn	mouse	1:100	DSHB
anti-Mega	mouse	1:50	Reinhard Schuh lab
anti-Trh	rat	1:100	Produced by N. Martín in Casanova lab
secondary antibodies			
name	generated in	Conc.	source/provided by
anti-goat	donkey	1:300	Life Technologies
anti-mouse	donkey	1:300	Life Technologies
anti-rabbit	donkey	1:300	Life Technologies
anti-rat	donkey	1:300	Life Technologies
fluorescent probe			
name	description	conc.	source/provided by
CBP	chitin binding probe with a fluorochrome	1:300	produced by N. Martín (New England Biolabs Protocol)

Abbreviations used: Conc., concentration; DSHB, Developmental Studies Hybridoma Bank.

### 3.1. *Drosophila* strains

*Drosophila melanogaster* strains were maintained and raised at 25°C under standard conditions. The following stocks were used: *UAS-Dia^CA^* (BDSC 27616); *UAS-Rab5^DN^* (kindly provided by M. González-Gaitán); *UAS-GFP* (kindly provided by J. Solon); *UAS-mCherryCAAX* (BDSC 59021); *btlGal4, UAS-SrcGFP* (from Llimargas's lab); *btlGal4, DE-cadGFP* (from M. Llimargas's lab); *btlGal4; btl::MoeGFP* (from J. Casanova's lab); *hsFLP^122^;btl::RFPMoe, btl FRT > y+>FRT Gal4* (kindly provided by M. Affolter); *cycA^C8LR1^/TM3* (BDSC 6627); *sdk^MB05054^* (BDSC 24603) and *dap^4^/CyO* (BDSC 6639).

Balancer chromosomes were used to follow the constructs of interest in the different chromosomes for the combinations and generation of recombinant strains. Transgene expression was achieved using the Gal4/UAS system [41] at 29°C, or at 25°C when specified.

### 3.2. Immunohistochemistry

Embryos were stained following standard protocols. Embryos were fixed in 4% formaldehyde (Sigma-Aldrich) in PBS1x-Heptane (1:1) for 10 min for Ecad staining and for 20 min for the rest. Embryos transferred to new tubes were washed in PBT-BSA blocking solution and shaken in a rotator device at room temperature. Embryos were incubated with the primary antibodies in PBT-BSA overnight at 4°C. Secondary antibodies diluted in PBT-BSA (and for the CBP staining) were added after washing and were incubated at room temperature for 2 h in the dark. Embryos were washed, mounted on microscope glass slides with Fluoromount-G (Southern Biotech) and covered with thin glass slides.

The following primary antibodies were used: rabbit anti-DSRF (1:500: produced by N. Martín in J. Casanova's lab); rat anti-Ecad (1:100; DCAD2, Developmental Studies Hybridoma Bank (DSHB)); goat anti-Ecad (1:600; Santa Cruz); mouse anti-Crb (1:10; Cq4, Developmental Studies Hybridoma Bank (DSHB)); mouse anti-Mega (1:50; kindly provided by Dr R. Schuh); goat anti-GFP (1:300; ABCAM); rabbit anti-GFP (1:300, Life Technologies); mouse anti-Sn (1:100; DSHB) and rat anti-Trh (1:100; produced by N. Martín in J. Casanova's lab). The following secondary antibodies were used: donkey anti-goat; anti-mouse; anti-rabbit; and anti-rat (1:300; Life Technologies).

### 3.3. Generation of tracheal cell clones

Tracheal cell clones were generated by combining the hsFlp/FRT and the Gal4/UAS systems to induce randomly generated cell clones, using the *btlGal4* driver. Agar plates containing embryos of 10–12 h after egg laying were heat-shocked in a water bath at 37°C. Two heat shocks for 45 min, with a 30 min break outside at room temperature, were performed. Embryos were kept at 25°C for 5–6 h until they were fixed and processed for immunostaining.

### 3.4. Image acquisition

Images from fixed embryos were taken using a Leica TCS-SPE confocal microscope, with the 20× and 63× immersion oil (1.40–0.60; Immersol 518F—Zeiss oil) objectives and additional zoom. Settings were adjusted for the different channels prior to image acquisition. Z-stack sections of 0.3–0.5 µm were acquired. The images were imported and processed using Fiji (ImageJ 1.49b). The images shown here are typically projections of confocal Z-stack sections, unless otherwise stated.

Embryos for live imaging were collected from agar plates after 10–12 h of incubation, dechorionated using 100% bleach and washed with PBS1×. Selection of the embryos at late st.13 was done using a fluorescent scope to identify the autofluorescence of embryo yolk. Embryos were lined up on a thin coverslip (22 × 50 µm Menzel Gläzer #0) with 10-S Voltalef oil (VWR) and covered with a stretch membrane (YSI membrane kit). Live imaging acquisition started typically at 30–40 min after mounting the sample.

Live image acquisition was performed in a Zeiss 780 LMS microscope (ZEN 2.1 software). The 63x (1.4 NA) oil immersion objective was used (Immersol 518F—Zeiss immersion oil), with maximization of the scanning area with the zoom options. Acquisition settings (dyes, laser intensity, scanning speed, bidirectionality, etc.) were adjusted and the photon counting detection operation mode was used. Z-stack time series were obtained in intervals of 30–90 s for two channels and intervals of 30 s for one channel. Recentring of the sample was done when needed during image acquisition. Z-stack time series were processed with Fiji (ImageJ 1.49b), projected along the *Z*-axis using the maximal intensity option, concatenated and adjusted with the StackReg plugin. Still images from the *in vivo* experiments are shown as Z-stack projections.

### 3.5. Quantifications and data analysis

Dorsal branches length was measured from the base of the dorsal branch (proximal part linked to the dorsal trunk) to the base of the tip cells (distal part), considering the Ecad signal. The length of the stalk cells was measured also considering the Ecad signal, from one intercellular AJ ring to the next one (electronic supplementary material, figure S1). Quantifications were performed in the dorsal branches of tracheal metameres 5–8. Dorsal branch diameter was measured considering the width of the tube stained with CBP on three points along the dorsal branch of tracheal metamere 5. These points were 15%, 50% and 85%, from the proximal to the distal part of the total dorsal branch extension (figure 5*a*). Note that lumen diameter is an approximation based on the length of the lateral area assuming a homogeneous ‘internal surface area’ in each tube. Measurements were performed tracing the paths using the freehand selection tool in Fiji (ImageJ 1.49b). The number of stalk cells forming a dorsal branch was obtained by counting the nuclei (by anti-Trh staining) and/or considering the data obtained in the quantification of the cell’ lengths.

Data from quantifications were imported and treated in Excel 14.7.1 and/or in GraphPad Prism 8.4.3, where graphics were finally generated. Graphics shown here are scatter dot plots, where bars indicate the mean and the standard deviation, and stacked bars. Quantifications in the text are also shown as mean ± s.d. Statistical analyses comparing the mean of two groups of quantitative continuous data were performed by unpaired two-tailed Student's *t*-test applying Welch's correction. Statistical analyses for multiple comparisons of quantitative continuous data were performed by Welch's ANOVA test followed by Dunnett's T3 *post hoc* test. Differences were considered significant when *p* < 0.05. Significant differences are shown in the graphics as: *p < 0.05, **p < 0.01, ***p < 0.001. ****p < 0.0001. n.s. means not statistically significant. Sample size (*n*) is provided in the figure legends.

## Supplementary Material

Fig S1.

## Supplementary Material

Fig S2.

## Supplementary Material

Fig S3.

## Supplementary Material

Fig S4.

## Supplementary Material

Movie 1

## Supplementary Material

Movie 2

## Supplementary Material

Movie 3

## Supplementary Material

Movie 4

## Data accessibility

This article has no additional data.
