# Supplementary figures and images for: Unravelling the distinct contribution of cell shape changes and cell intercalation to tissue morphogenesis: the case of the Drosophila trachea
Source: Open Biol. 2020 Nov 25;10(11):200329. doi: 10.1098/rsob.200329 (PMC7729023; doi:10.1098/rsob.200329)

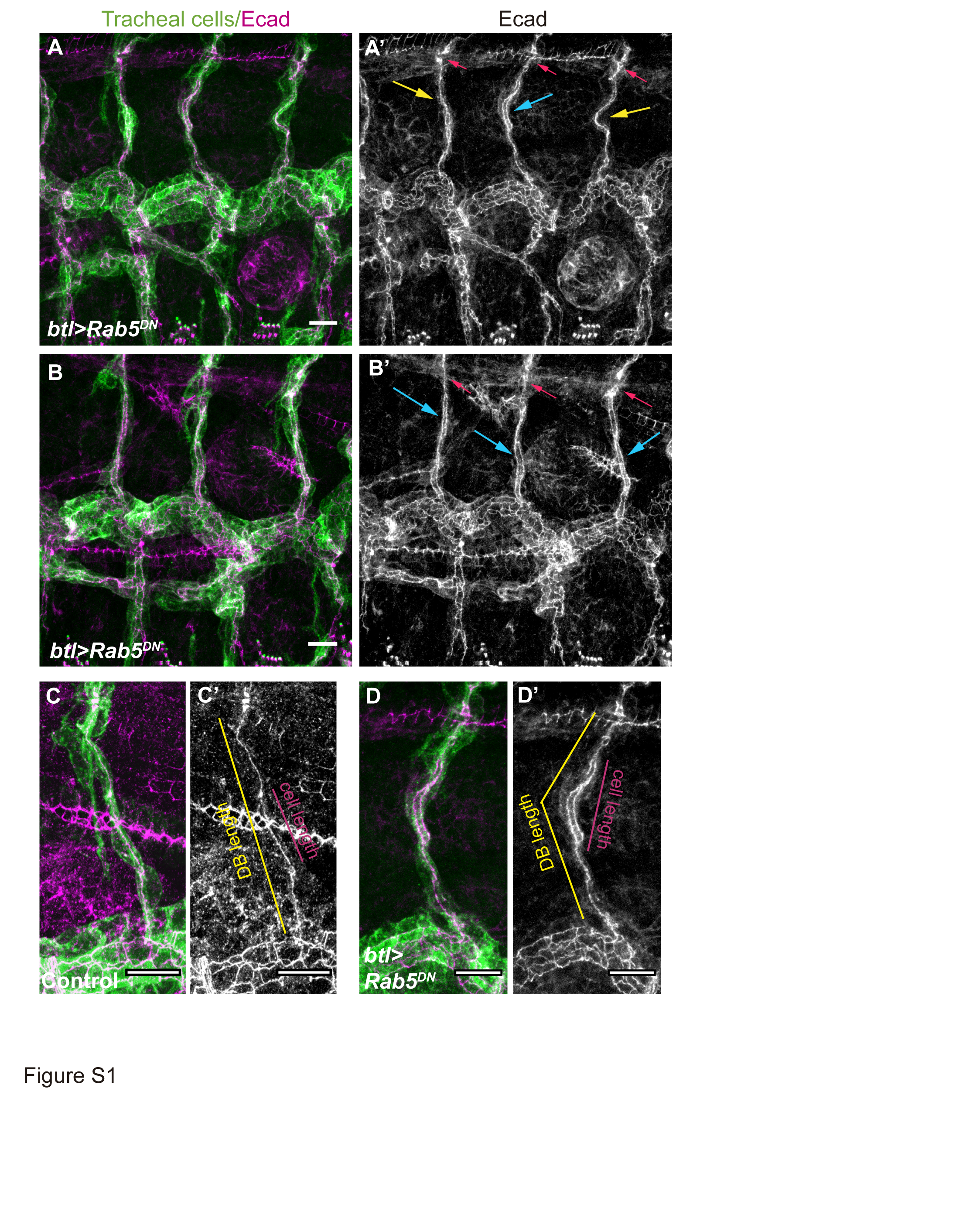

Supplement: Fig S1. [file rsob200329supp1.tif]

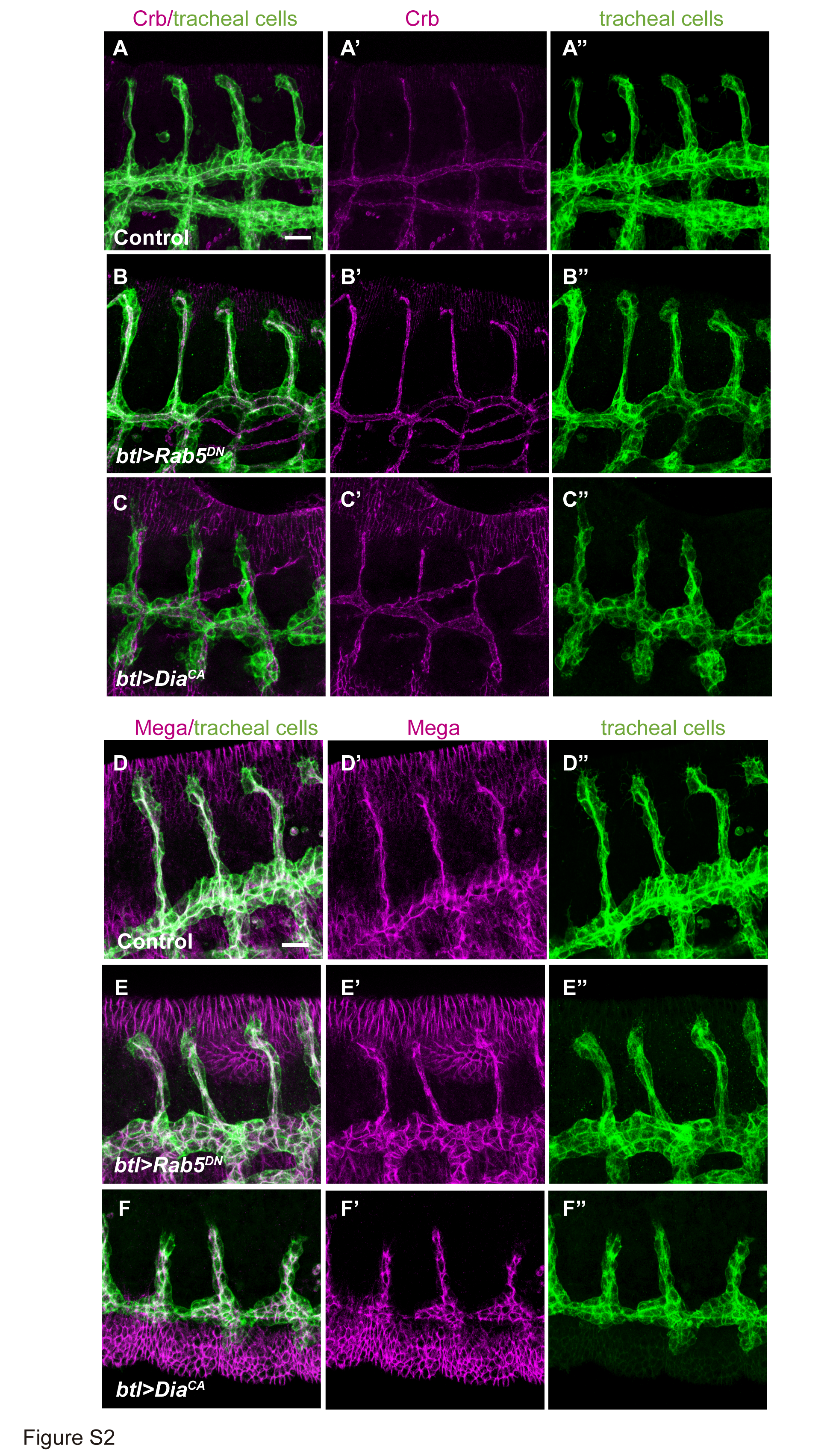

Supplement: Fig S2. [file rsob200329supp2.tif]

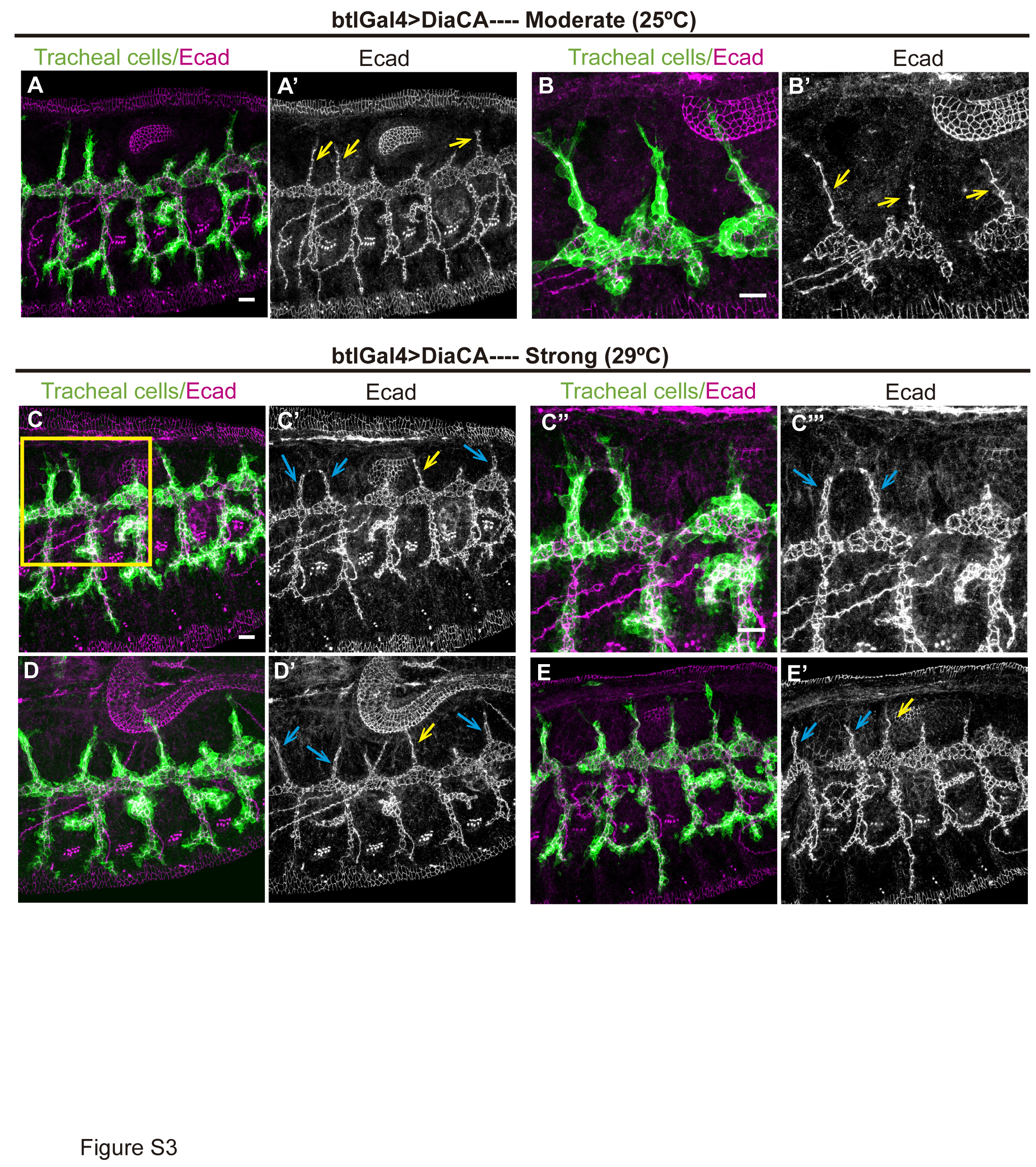

Supplement: Fig S3. [file rsob200329supp3.tif]

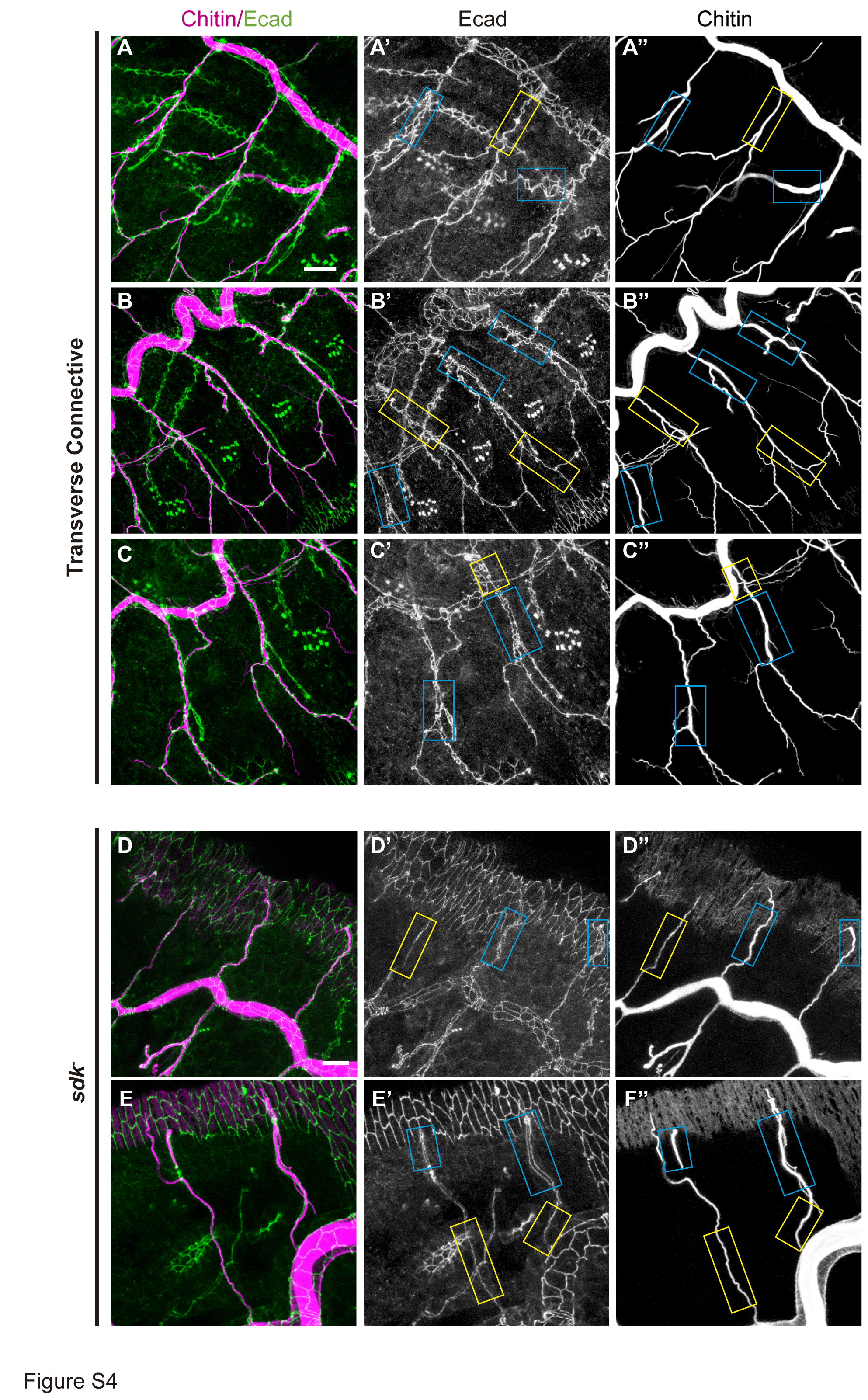

Supplement: Fig S4. [file rsob200329supp4.tif]
